# Adaptive-size dictionary learning using information theoretic criteria for image reconstruction from undersampled k-space data in low field magnetic resonance imaging

**DOI:** 10.1186/s12880-020-00474-3

**Published:** 2020-06-29

**Authors:** Emmanuel Ahishakiye, Martin Bastiaan Van Gijzen, Julius Tumwiine, Johnes Obungoloch

**Affiliations:** 1 Department of Computer Science, Kyambogo University, P.O. Box 1, Kyambogo, Kampala, Uganda; 2 Department of Biomedical Sciences and Engineering, Mbarara University of Science and Technology, P.O.Box 1410, Mbarara, Uganda; 3 Delft Institute of Applied Mathematics, Delft University of Technology, Mekelweg 4, 2628 CD Delft, The Netherlands; 4 Department of Mathematics, Mbarara University of Science and Technology, P.O.Box 1410, Mbarara, Uganda

**Keywords:** Compressed sensing, Dictionary learning, Image reconstruction, Information-theoretic criteria, Low-field MRI

## Abstract

**Background:**

Magnetic resonance imaging (MRI) is a safe non-invasive and nonionizing medical imaging modality that is used to visualize the structure of human anatomy. Conventional (high-field) MRI scanners are very expensive to purchase, operate and maintain, which limit their use in many developing countries. This study is part of a project that aims at addressing these challenges and is carried out by teams from Mbarara University of Science and Technology (MUST) in Uganda, Leiden University Medical Center (LUMC) in the Netherlands, Delft University of Technology (TU Delft) in the Netherlands and Pennsylvania State University (PSU) in the USA. These are working on developing affordable, portable and low-field MRI scanners to diagnose children in developing countries with hydrocephalus. The challenges faced by the teams are that the low-field MRI scanners currently under development are characterized by low Signal-to-Noise Ratio (SNR), and long scan times.

**Methods:**

We propose an algorithm called adaptive-size dictionary learning algorithm (AS-DLMRI) that integrates information-theoretic criteria (ITC) and Dictionary learning approaches. The result of the integration is an adaptive-size dictionary that is optimal for any input signal. AS-DLMRI may help to reduce the scan time and improve the SNR of the generated images, thereby improving the image quality.

**Results:**

We compared our proposed algorithm AS-DLMRI with adaptive patch-based algorithm known as DLMRI and non-adaptive CSMRI technique known as LDP. DLMRI and LDP have been used as the baseline algorithms in other related studies. The results of AS-DLMRI are consistently slightly better in terms of PSNR, SNR and HFEN than for DLMRI, and are significantly better than for LDP. Moreover, AS-DLMRI is faster than DLMRI.

**Conclusion:**

Using a dictionary size that is appropriate to the input data could reduce the computational complexity, and also the construction quality since only dictionary atoms that are relevant to the task are included in the dictionary and are used during the reconstruction. However, AS-DLMRI did not completely remove noise during the experiments with the noisy phantom. Our next step in our research is to integrate our proposed algorithm with an image denoising function.

## Background

Magnetic resonance imaging (MRI) is a technique that uses magnetic fields to create tomographic images without exposure to radiation. The use of MRI technology in medicine provides noninvasive and nonionizing visualization of the structure of human anatomy [1]. The MRI systems are classified according to the strength of the magnetic field they produce (in Tesla): Ultra high field > 3 T, high field (1 - 3 T), midfield (0.5 - 1 T), low field (0.1–0.5 T) and ultra-low field < 0.1 T [2]. The conventional MRI scanners (high-field) are very expensive to purchase, operate and maintain and are therefore often out of reach in developing countries. These limitations can be overcome by developing affordable, portable and low-field MRI scanners that can be used to diagnose conditions that do not require a high resolution, such as hydrocephalus [3].

Moreso, MRI scanners have long acquisition time, and this means that MRI is a slow imaging modality. The challenge of long acquisition time in MRI can be addressed by using either hardware or software approach. The hardware approach uses a multichannel parallel MRI but it is economically expensive and requires considerable time in development [4]. The software approach uses efficient algorithms majority of which are based on compressed sensing (CS) [5]. Using CS based reconstruction algorithms guarantees improvement in image quality and also improvement in acquisition speed because images/signals can be accurately recovered using fewer measurements than mandated by the traditional Nyquist sampling [6].

Recently, CS theory has been applied to MRI demonstrating high quality reconstructions from fewer measurements than those mandated by the traditional Nyquist sampling [6]. CS is defined as “the process of efficiently acquiring either a sparse signal directly or a signal which is compressible in some known domain” [4]. Using CS theory, a sparse signal *x* ∈ C^*n*^can be acquired using a measurement matrix *A* ∈  C^*m x n*^ with *m* > *n* so that the measured signal *y* = *Ax* Then, *x* can be reconstructed from *y* ∈  C^*m*^ if both x and A are sparse or at least compressible in some transform domain [4] by solving the minimization problem in Eq. 1 below.

1 $$ {\min}_x{\left\Vert \mathrm{x}\right\Vert}_0\mathrm{subject}\ \mathrm{to}\ y= Ax, $$

where║x║_0_ indicates the nonzero coefficients in *x*. Equation (1) is non-convex and is solved by replacement of *ℓ*_0_– minimization with *ℓ*_1_– minimization, described as;

2 $$ {\min}_x{\left\Vert \mathrm{x}\right\Vert}_1\;\mathrm{subject}\ \mathrm{to}\ y= Ax, $$

where ║x║_1_ = ${\sum}_{i=1}^n\mid {x}_i\mid$. *ℓ*_1_– minimization is a convex problem that can be solved using a linear programming approach, a primal-dual interior-point method, and also using greedy algorithms such as Orthogonal Matching Pursuit (OMP) [7]. Greedy algorithms are fast, simpler and suitable for hardware implementation [4]. Sometimes the measured signal y may contain noise from the surrounding environment, we can then relax the equality constraint and rewrite Eq. 2 as shown in Eq. 3.

3 $$ {\min}_x{\left\Vert \mathrm{x}\right\Vert}_1\;\mathrm{subject}\ \mathrm{to}\ {\min}_x{\left\Vert y-\mathrm{Ax}\right\Vert}_2\le \varepsilon, $$

where *ε* is a positive constant indicating the noise level.

MRI inherently fulfills all the requirements for compressed sensing theory for image reconstruction because images are sparse in wavelet domain and there exist a strong incoherence between the Fourier and the wavelet domain [8]. However, non-adaptive CS techniques are usually limited in typical MR images to 2.5–3 fold undersampling. They also result into many undesirable artifacts and loss of features. Also, explicit k-space interpolation in CSMRI [8] leads to poor reconstructions due to the lack of local structure in k-space [6]. To overcome the challenges of non-adaptive CS techniques, Ravishankar & Bresler [6] proposed an adaptive patch-based framework known as DLMRI [6] for simultaneously learning the dictionary and reconstructing the image from highly undersampled k-space data. In DLMRI [6], the researchers learnt an image-patch dictionary from a small number of k-space samples. Adaptive CS techniques can sparsify images better compared to non-adaptive CS techniques [8] since they are learnt for the particular image instance or class of images. The shift from global image sparsity to patch-based sparsity is captivating since patch based dictionaries can capture local image features effectively, and can potentially remove noise and aliasing artifacts in CSMRI [8] without sacrificing resolution. The advantage of DLMRI [6] framework is that it increases the reconstruction accuracy even in the absence of the reference image by directly adapting to the image content. However, determining the optimal dictionary size that is satisfactory for a given input signal in DLMRI [6] and other adaptive transforms is currently a challenge.

Determining the fitting dictionary size is very crucial in image processing tasks. This is due to the fact that large dictionaries influence both the dictionary learning process and the representation speed. Large dictionaries may lead to overfitting as the number of dictionary atoms increases and therefore the dictionary becomes less representative of the input signal. On the other side, a dictionary with fewer atoms may lead to the loss of some image features since some of the important dictionary atoms are left out and this has an impact on the quality of the final image. According to literature, the common method is to impose a representation error and then selecting a dictionary size that gives the minimum error [9]. The algorithms using this technique use sparse coding and dictionary update where atoms are added or removed during the dictionary learning (DL) process. The techniques that are currently available works as follows; (1) an algorithm starts with a dictionary with a small number of atoms and then new atoms are added during the DL iterations that are able to minimize the error, or (2) an algorithm starts with a dictionary with a large number of atoms and then less significant ones are removed during the DL iterations [9]. In this study, we propose an image reconstruction algorithm that adapts the dictionary size using information-theoretic criteria (ITC). Our proposed framework combines the advantages of adaptive patch-based dictionaries with those of optimal dictionary size resulting into high quality reconstructions. We hypothesize that by using an adaptive-size dictionary learning we can increase the performance on image reconstruction over previous methods.

### Context of our study

Our research is part of a larger programme funded by the Dutch organization NWO-WOTRO to stimulate research that addresses the Sustainable Development Goals (SDGs). By developing low-cost MRI scanners we aim to contribute to SDG 3: “Ensure healthy lives and promote well-being for all at all ages”. Our project is carried out by teams from Mbarara University of Science and Technology (MUST) in Uganda, Leiden University Medical Center (LUMC) in the Netherlands, Delft University of Technology (TU Delft) in the Netherlands and Pennsylvania State University (PSU) in the USA and aims to develop low-field MRI scanners and image processing algorithms in particular for low-resource settings. The low-field MRI systems under development are characterized by a low signal-to-noise ratio, and this has a very big impact on the quality of the final image [10]. Also, it takes a long time to acquire an image (takes more than 16 min to scan an object). With the algorithm that is described in this paper, we aim to eliviate these drawbacks of low-field MRI. Our project is ongoing and we expect to start clinical trials in the first half of 2021. The prototype that has been developed by the LUMC will be shipped for testing at MUST in Uganda once total lockdowndue to Covid19 is lifted.

## Methods

### Dictionary learning (DL)

Dictionary learning problem is the search for optimal dictionaries for a specific set of training signals. The technique that is commonly used in DL problems is sparse coding. Sparse coding is a technique of finding a representation of a given signal with a smaller number of significant coefficients. The study [6] noted that “Non adaptive CSMRI techniques are limited by the degree of undersampling at which they can still give clinically useful reconstructions”. Adaptive dictionaries lead to higher sparsities and hence to potentially higher undersampling factors in CSMRI. DL is a key to adapting dictionaries of the data required for CSMRI reconstructions.

Given an image *x* ∈ C^*n*^, *x*_*ij*_ ∈ C^*n*^ is the vector representation of a square 2D image patch of √*n x* √ *n* pixels indexed by the location of its top-left corner (*i*, *j*) in the image. *D* ∈ C^*n X K*^ is used to represent the image patched dictionary with *K* atoms each with n-vector corresponding to a √*n x* √ *n* elemental patch. In Compressed Sensing MRI, it is assumed that each patch *x*_*ij*_ can be approximated as a linear combination *Dα*_*ij*_ of the dictionary atoms, where *α*_*ij*_ ∈ C^*K*^ is sparse. *D* can be predefined (such as overcomplete wavelets) or learned. Each column in the dictionary (*D*) is called an atom and *D* has *K* atoms. When the number of atoms equals the number of the patch (*K* = *n*), *D* is said to be the basis (complete dictionary). The dictionary *D* is “overcomplete” in case *K* > *n* with the latter being a typical assumption for a sparse dictionary learning problem. The issue of a complete dictionary is not considered because it does not provide any improvement from a representational point of view. Therefore during this study, we considered a case of *K* > *n*.

The DL problem aims at solving the following optimization problem:

4 $$ \underset{D,\Gamma}{\min }{\sum}_{ij}{\left\Vert {\mathrm{R}}_{\mathrm{ij}}\mathrm{x}\hbox{-} {\mathrm{D}\upalpha}_{\mathrm{ij}}\right\Vert}_2^2\mathrm{subject}\ \mathrm{to}\ {\left\Vert {\upalpha}_{\mathrm{ij}}\right\Vert}_0\le {T}_0\forall \mathrm{i},\mathrm{j}, $$

Where R_ij_ ***∈*** C^*n X P*^ represents the operator that extracts the patch x_ij_ from x as x_ij_ = R_ij_x. The ℓ_0_ quasi norm (║α_ij_║_0_) is used to encode the sparsity of the patch representation and *T*_*0*_ is the required sparsity level. *Г* is used to denote the set {*α*_*ij*_}*ij* of the sparse representations of all the patches. The optimization of a dictionary *D* and sparse coefficients *Г* is a nonconvex problem. This problem is related to sparse coding that requires finding a sparse code and also the dictionary for sparse presentation has to be estimated simultaneously. The easiest way of solving such a problem is to solve separately sparse code and the dictionary and iteratively alternating their solutions until convergence. There are two major efficient algorithms to learn dictionaries that utilize variants during the iterative optimization strategy and these are the K-SVD [11] and the method of optimal directions (MOD) [12]. It is noted from the literature that K-SVD converges with fewer iterations than MOD. For more details, refer to [11, 12].

### Information-theoretic criteria (ITC)

The ITC implementation that we used during this study was adapted from [13]. We used ITC for assessing the adequacy of a model for dictionary learning by combining its goodness of fit (root mean square error) with its complexity. For a given training signal *Y* ∈ C^*mxN*^, the signal to be reconstructed *X* ∈ C^*nxN*^, the sparsity s, and the dictionary *D* ∈ C^*mxn*^, the root mean square error and complexity are expressed as shown in Eqs. 5 and 6 as follows;

5 $$ Root\kern0.17em mean\kern0.17em square\kern0.17em error(RMSE)=\frac{1}{\sqrt{Q}}{\left\Vert Y- DX\right\Vert}_F\;\mathrm{where}\ Q= mN $$

where m and N are the dimensions of the training signal.

The complexity depends on several parameters and it is defined as follows:

6 $$ complexity\ (P)= sN+\left(m-1\right)n, $$

where the first term (*sN*) corresponds to the number of nonzero elements in X while the second term corresponds to the number of independent elements of the dictionary [13].

Basing on the Eqs. 5 and 6, two ITC formulations are computed as shown in Eqs. 7 and 8. The formulation in Eq. 7 is known as Extended Bayesian Information Criterion (EBIC), while that in Eq. 8 is known as Extended Renormalized Maximum Likelihood (ERML). Both formulations are capable of determining the optimal dictionary size during medical image reconstruction tasks. EBIC and ERML are shown in the Eqs. 7 and 8 respectively;

7 $$ EBIC=2\mathit{\log}\  RMSE+\frac{\mathit{\log}(Q)}{(Q)}P+\frac{2N}{(Q)}\mathit{\log}\ \left(\genfrac{}{}{0pt}{}{n}{s}\right), $$

where the first two terms are standard ITC terms and the third term accounts for all possible positions of the non-zero entries in the matrix X.

8 $$ ERML=\left(Q-P\right)\mathit{\log}\frac{RMSE^2}{\left(Q-P\right)}+P\;\mathit{\log}\frac{{\left\Vert DX\right\Vert}_F^2}{Q.P}+\mathit{\log}\left[P\left(Q-P\right)\right]+2N\;\mathit{\log}\left({}_s^n\right). $$

All the experiments during this study use the EBIC approach. For more details, refer to [13].

### Candidate dictionaries

Using the ITC approach, several candidate dictionaries are needed from which a selection is made to a dictionary with the minimum ITC value. Usually, a single instance of the learning algorithm is run and therefore a single dictionary is available. The only possibility available is to compare smaller dictionaries made of a subset of the atoms, which requires large number of possible combinations. Their numbers can be reduced by ordering the atoms basing on their importance in representations. Given that,

9 $$ DX={\sum}_{j=1}^n{d}_j{x}_j^T, $$

where *x*_*j*_^*T*^ is the j^th^ row of *X*, we sort the atoms in decreasing order of their “power” (importance),

10 $$ P\left({d}_j\right)={\left\Vert {x}_j^T\right\Vert}_{2\cdot } $$

After the operations in Eqs. 9 and 10, we obtain a sorted dictionary, *D*. During selection, dictionaries *D*_*h*_ ∈ C^*mxh*^ are considered that are made of *h* ≤ *n* atoms of D. This shows that there are atmost *n* candidate dictionaries. Imposing a lower bound, *n*_min_ helps to eliminate small dictionaries that are not useful and therefore, we have *n* ≥ *n*_min_. Also, *n*_*min*_ = *m* can be chosen. For more details, refer to [13].

### Image reconstruction formulation in MRI

The image reconstruction formulation used during this study was adapted from Ravishankar and Bresler [6]. Consider the formulation below,

11 $$ \left(\mathrm{F}0\right)\underset{x,D,\Gamma}{\min }{\sum}_{ij}{\left\Vert {\mathrm{R}}_{\mathrm{i}\mathrm{j}}\mathrm{x}\hbox{-} {\mathrm{D}\upalpha}_{\mathrm{i}\mathrm{j}}\right\Vert}_2^2+\mathrm{w}{\left\Vert {\mathrm{F}}_{\mathrm{u}}\mathrm{x}\hbox{-} \mathrm{y}\right\Vert}_2^2\kern0.36em \mathrm{subject}\ \mathrm{to}\ {\left\Vert {\upalpha}_{\mathrm{i}\mathrm{j}}\right\Vert}_0\le {T}_0{\forall}_{\mathrm{i},\mathrm{j},} $$

where the first term captures the quality of sparse approximations of the image patches with respect to the dictionary *D* while the second term enforces data fidelity in the k-space. The weight *w* is defined as *w* = (⋋/*δ*), where ⋋ is a positive constant. The formulation has been proven to be more robust to noise [6]. This formulation was proposed by [6], it is capable of designing an adaptive dictionary, and can also be used to reconstruct an underlying image from k-space measurements. It can potentially remove aliasing and noise while learning the local image features effectively. However, eq. (11) is NP-hard and nonconvex even when the ℓ_0_ quasi norm is relaxed to ℓ_1_ quasi norm. it was solved using a two-step procedure in DLMRI [6] as explained below.

i) The first step (dictionary learning step): in this step, x remained fixed, and the dictionary and sparse representations of the patches are jointly learnt. The corresponding subproblem is:

12 $$ \left(\mathrm{F}1\right)\underset{D,\Gamma}{\min }{\sum}_{i,j}{\left\Vert {\mathrm{R}}_{\mathrm{i}\mathrm{j}}\mathrm{x}\hbox{-} {\mathrm{D}\upalpha}_{\mathrm{i}\mathrm{j}}\right\Vert}_2^2\kern0.24em \mathrm{subject}\ \mathrm{to}\ {\left\Vert {\mathrm{d}}_{\mathrm{k}}\right\Vert}_2=1{\forall}_{\mathrm{k}},{\left\Vert {\upalpha}_{\mathrm{i}\mathrm{j}}\right\Vert}_0\le {T}_0{\forall}_{\mathrm{i},\mathrm{j},} $$

In DLMRI, the dictionary D was learnt using the K-SVD algorithm [11] and once the dictionary is learned, sparse coding was performed on all patches to determine the α_ij_ using OMP [7].

ii) The second step (updating the reconstruction): in this step, the formulation (F0) is solved with the fixed dictionary and sparse representations. The corresponding subproblem is:

13 $$ \left(\mathrm{F}2\right)\underset{x}{\min }{\sum}_{ij}{\left\Vert {\mathrm{R}}_{\mathrm{ij}}\mathrm{x}\hbox{-} {\mathrm{D}\upalpha}_{\mathrm{ij}}\right\Vert}_2^2+\mathrm{v}{\left\Vert {\mathrm{F}}_{\mathrm{u}}\mathrm{x}\hbox{-} \mathrm{y}\right\Vert}_2^2, $$

Equation (12) is a least-squares problem which was solved in DLMRI [6] using the Eq. (14) below.

14 $$ Fx\left({k}_x,{k}_y\right)=\left\{\begin{array}{c}M\left({k}_x,{k}_y\right),\kern0.5em \left({k}_x,{k}_y\right)\notin \varOmega \\ {}\frac{M\left({k}_x,{k}_y\right)+v\ {M}_o\left({k}_x,{k}_y\right)}{1+v},\kern0.5em \left({k}_x,{k}_y\right)\in \varOmega \end{array}\right. $$

where *Fx*(*k*_*x*_, *k*_*y*_) represents the updated value at location $\left({k}_x,{k}_y\right),{M}_0=F{F}_u^Hy$ represents the zero-filled k-space measurements, and *Ω* represents the subset of k-space that has been sampled. The reconstruction, x, is then obtained by IFFT of *Fx*. For more details, refer to [6].

### The proposed algorithm

**Fig. 1:**
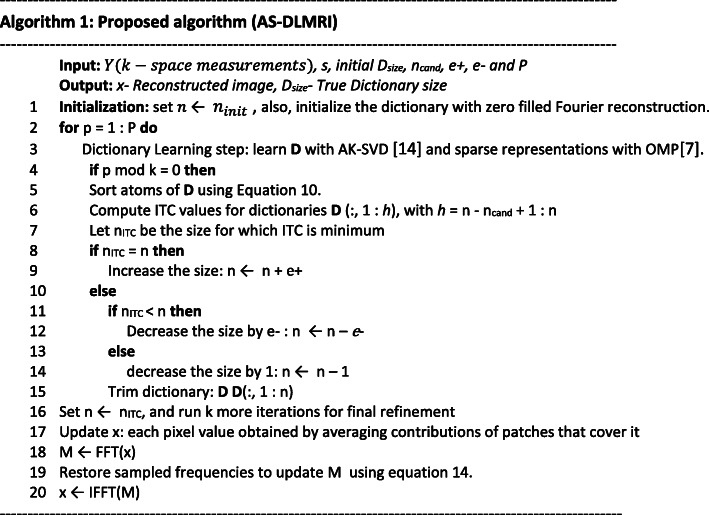
Our proposed Adaptive-Size Dictionary Learning MRI (AS-DLMRI) Algorithm

In this paper, we propose an adaptive-size dictionary learning algorithm (abbreviated as AS-DLMRI), an extension of DLMRI [6]. In the subsequent sections, we will be AS-DLMRI uses an ITC approach to determine anoptimal dictionary size. As with DLMRI, AS-DLMRI also have two steps namely, the dictionary learning step and the reconstruction update step. The two steps are explained in details as follows;

i. In the first step (the dictionary learning step), x is fixed, and then the dictionary and sparse representations of the patches are jointly learned. we initialize the dictionary with the left singular vectors of the training data. Also, our proposed algorithm assumes an initial dictionary size (*n*_*init*_). We learned the dictionary using the Approximate K-SVD (AK-SVD) algorithm [14], and sparse coding using OMP [7]. It is during this step that the candidate dictionaries are sorted in the order of importance using ITC as explained above. To avoid overcomputation, only a small number of candidates *n*_*cand*_ is considered. We also consider *n*_*ITC*_ as the minimum size of the ITC value among the *n*_*cand*_. We then consider *n*_*ITC*_ as an the indicator in which *n* evolves. Then, the following three tests are performed. (1) if *n*_*ITC*_ is much smaller than the current dictionary size *n*, we decrease the size by *e*-, (2) if *n*_*ITC*_ is slightly smaller than the current dictionary size *n*, we decrease the size by one, and (3) if *n*_*ITC*_ equals to *n*, we increase the size by *e* +. During our experiments, we used 5 for the values *e*- and *e* +. Incase new atoms are required, a random technique is used to generate them. After P iterations, the value of *n*_*ITC*_ is considered as the true dictionary size. For final refinement, k more iterations are required.
ii. In the second step (Updating the reconstruction), x is updated but the dictionary and the sparse representations are fixed. This step involves solving a least squares problem, and we also used eq. 14 to solve it. The summary of our proposed algorithm is summarized in Fig. 1. Below.

### Data sources

**Fig. 2:**
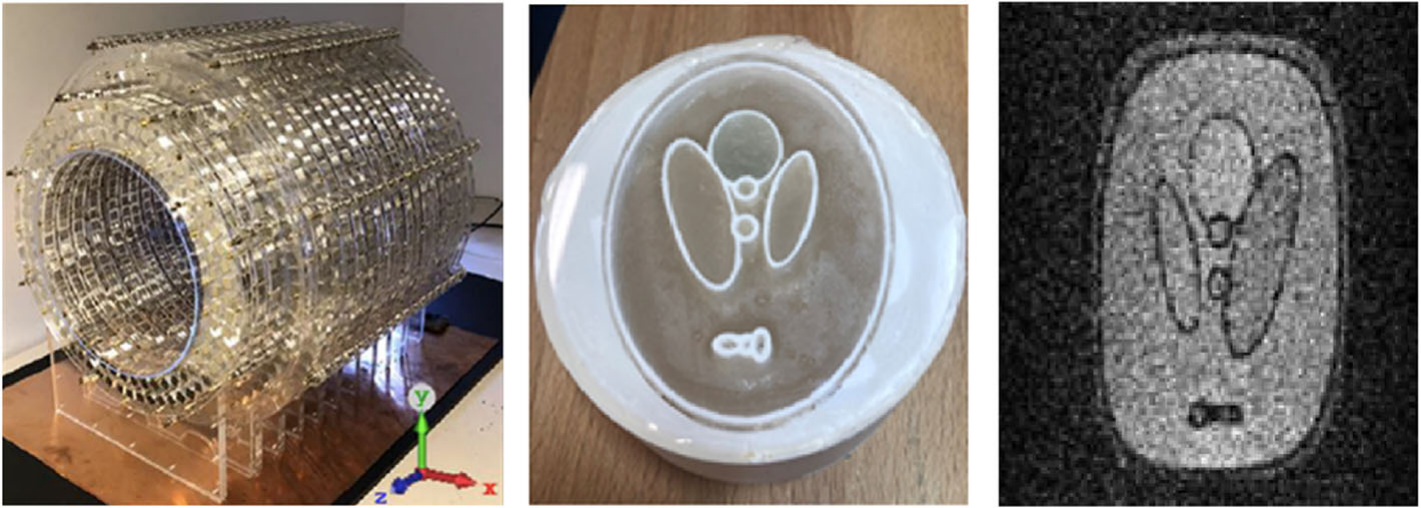
(Left) A low-cost portable MRI at LUMC, (Middle) The 3D printed phantom (Shepp Logan) and (Right) The image of the 2D phantom acquired using the Low-Field MRI scanner on the left

The measured image that is used in this study was obtained from the low-field MRI system that is currently under development at Leiden University Medical Center (LUMC). Figure 2 shows this low-field MRI scanner [15] and a 2D phantom image [16]. The 3D-printed physical phantom displayed in Fig. 2 (middle) is modeled after the classical Shepp-Logan phantom. It is 70 mm wide, 90 mm tall, 35 mm thick and filled with agar gel. The Image was acquired using a non-selective single-slice spin-echo sequence with the following parameters: Field of view: 128 × 128 mm, Acquisition matrix: 128 × 128, TR/TE: 500 ms/10 ms, scan duration: 2 min 4 s. In addition to this measured image, an MRI brain image obtained from [6] was used during the experiments. Further details on the low-field MRI scanners that are currently under development can be obtained from [2, 15, 16].

### Parameter selection

During our experiments, our algorithm was initialized with a zero-filled Fourier reconstruction. The images used in all the experiments were converted to overlapping patches of size √*n x* √ *n* . During this study, we used *n* = 36. It is from these image patches that we initialized data for dictionary learning. Left side vectors of the image patches were used as our initial dictionary. During the experiments, the following parameters were used; sparsity level, s is 5; DL iterations varied from 5 to 20; minimum considered dictionary size, nmin is 64; nminus is 5 (how much n decreases); nplus is 5 (how much n grows); ncand is 20 (number of size candidates for ITC); itc_index = 13 (ITC codes (11 for EBIC, 13 for ERNML)), K = n = 36, sparsity *τ*_0_ = 6, *γ* = 140, overlap stride *r* = 1. The matlab implementation of DLMRI [6] and non-adaptive CSMRI technique known as LDP by Lustig et al. [8] are both available, and we used the built in settings for both algorithms in our experiments.

### The performance metrics used

During this study, we adopted four performance metrics that have been used by other researchers in related studies. The four metrics are Peak Signal to Noise Ratio (PSNR), Signal to Noise Ratio (SNR), high-frequency error norm (HFEN) [6, 17] and reconstruction time. PSNR (measured in decibels-dB) is computed as the ratio of the peak intensity value of the reference image to the root mean square (RMS)reconstruction error relative to the reference image. This metric is considered as the image quality measure and has been used a lot in image compression and denoising tasks. HFEN was used to quantify the quality of reconstruction of edges and fine features. A rotationally symmetric LoG filter was used to capture edges. HFEN was then computed as the norm of the result obtained by LoG filtering the difference between the reconstructed and reference images. However, the parameters we used do not represent perceptual visual quality which can only be assessed by human visual observer studies accurately.

## Results

**Fig. 3:**
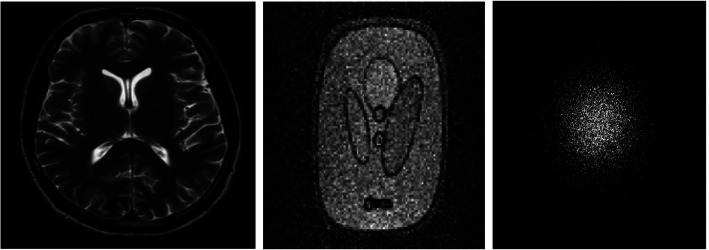
(Left) MRI image from [6], (Middle) phantom image from [15], and (Right) a sampling mask in k-space with 20 fold undersampling from [6]

In this section, we present the performance of our proposed algorithm AS-DLMRI. We compared the performance of our proposed algorithm with an adaptive patch-based dictionary learning technique known as DLMRI by Ravishankar & Bresler [6] and the leading non-adaptive CSMRI technique known as LDP by Lustig et al. [8]. DLMRI and LDP were selected because they have been widely used in the literature as the benchmark algorithms in related studies. Also, All the implementations were coded in Matlab 2017a. Also, all the experiments were performed with an intel core i7 8th generation CPU at 1.80 GHZ (8 CPUs) and 16 GB memory, employing a 64 bit windows 10 operating system. The Matlab implementation of both DLMRI and LDP are available from authors’ websites. For more details, refer to [6] for DLMRI and [8] for LDP. We used an MRI image from [6] and a phantom image from a low field MRI scanner from [16]. We also used undersampling masks with several downsampling factors from [6]. Figure 3 shows an MRI image, a phantom image and one of the sampling masks that we used during this study: a sampling mask in k-space with 20 fold undersampling.

### Experiment on reconstruction visual quality

**Fig. 4:**
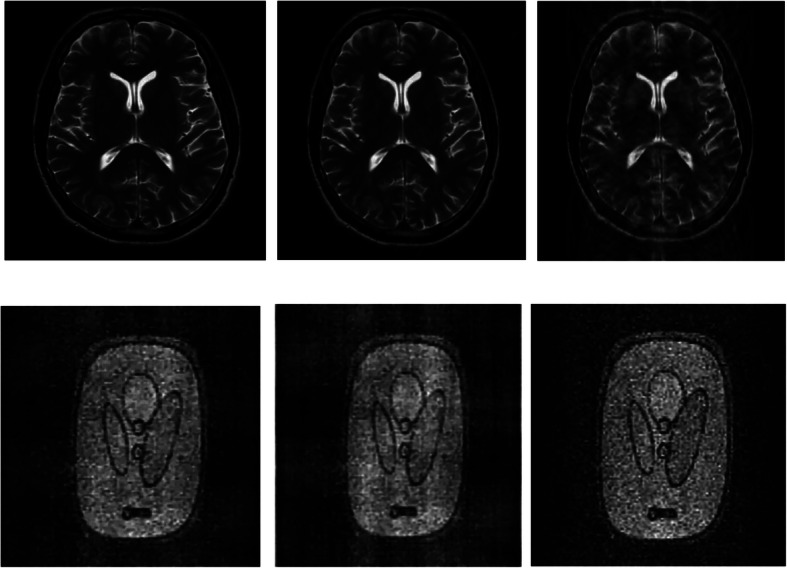
Experiment on reconstruction visual quality. (Top row) (left)- reconstruction of an MRI image using our proposed algorithm AS-DLMRI, (middle)-reconstruction using DLMRI and (right)-reconstruction using LDP. (Bottom row) (left)- Reconstruction of a phantom image using AS DLMRI, (middle)-reconstruction using DLMRI and (right)-reconstruction using LDP

In this section, we present the results of one of the several experiments that we did demonstrating the visual quality of the reconstructed images using AS-DLMRI, DLMRI and LDP. For the results shown in Fig. 4 below, we used a sampling mask in k-space with 10 fold undersampling, and the number of iterations was fixed to ten. It was noted that the visual quality for the images reconstructed by AS-DLMRI and DLMRI were almost identical, and therefore no significance visual difference between the images reconstructed by both algorithms. However, we noted a significance difference in visual quality of AS-DLMRI and LDP. We did other experiments with different undersampling masks and altering the number of iterations for dictionary learning, and we observed that AS-DLMRI and DLMRI produced almost identical visual quality results with a significance difference in visual quality when compared to LDP.

### Experiment on PSNR, HFEN and iteration number

**Fig. 5:**
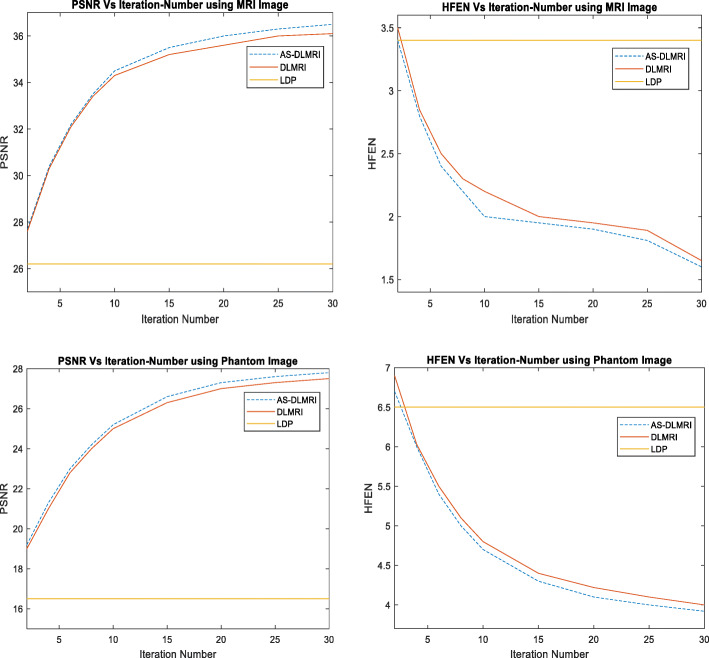
Experiment on PSNR, HFEN and Iteration Number. (Top) Experiment using an MRI image, (left) PSNR vs iteration number, (right) HFEN vs iteration number. (Down) Experiment using a Phantom image, (left) PSNR vs iteration number, (right) HFEN vs iteration number

Using the above images and the sampling mask, we did an experiment to determine how PSNR in each of the images changes as the number of iteration increases. It was observed as shown in Fig. 5 below that the PSNR with AS-DLMRI and DLMRI steadily increased as the number of iterations increased. However, AS-DLMRI had a slightly higher PSNR when compared to DLMRI. Also, It was observed in both AS-DLMRI and DLMRI that there is a very small difference between the PSNR when the number of iterations was 25 and PSNR when the number of iterations was 30. We therefore conclude that the number of iterations for the practical implementations of AS-DLMRI is in the order of 25 for sufficient quality. In comparison with LDP, AS-DLMRI produced better PSNR which steadily increased as the number of iterations increased, while for LDP, the value of PSNR remained constant as shown in Fig. 5 below. Higher values of PSNR indicates that AS-DLMRI reconstructs high quality images when compared with DLMRI and LDP. For HFEN, the value drastically decreased both for AS-DLMRI and for DLMRI but it remained constant for LDP. HFEN is lower for AS-DLMRI than for DLMRI and LDP in all our experiments indicating the superior performance of AS-DLMRI in capturing edges and fine features.

### Experiment on PSNR, HFEN and Undersampling factor

**Fig. 6:**
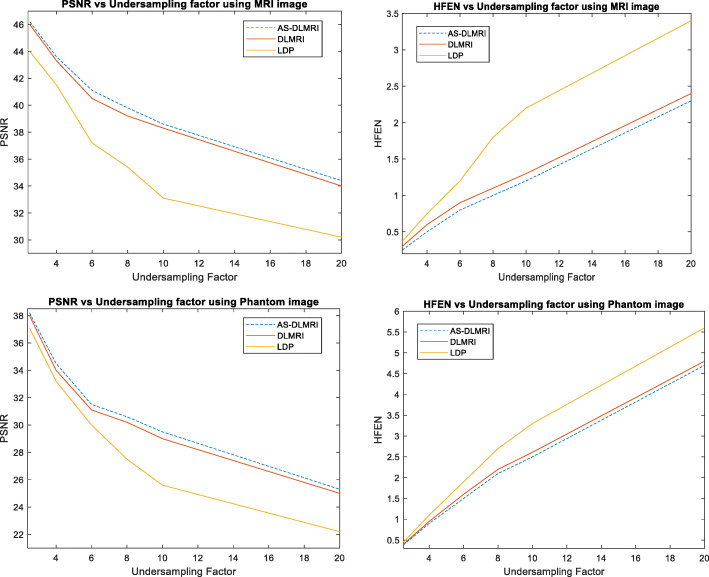
Experiments on PSNR, HFEN and Iteration Number. (Top) (left) PSNR vs undersampling factor using MRI image, (right) HFEN vs undersampling factor using MRI image, (Down)(left) PSNR vs undersampling factor using phantom image, and (right) HFEN vs undersampling factor u using phantom image

In this section, we discuss the results of our experiment in terms of PSNR, HFEN for different undersampling factors when the number of iterations was fixed to ten. It was observed as shown in the Fig. 6 below that PSNR reduces drastically with AS-DLMRI, DLMRI and LDP as the value of the undersampling mask in k-space increases. It was noted that AS-DLMRI produced a relatively high values of PSNR with different undersampling factors when compared to DLMRI and LDP. On the otherhand, the value of HFEN increases as the undersampling factor increases. It was noted as shown in Fig. 6 that AS-DLMRI had lower HFEN values with all the undersampling values.

### Experiment on SNR with Undersampling masks and iteration number

**Fig. 7:**
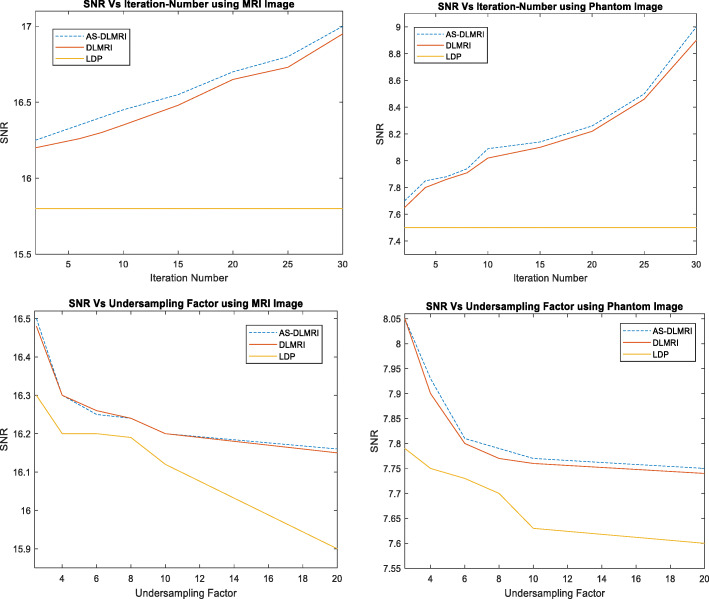
Experiments on SNR. (Top) (left) SNR vs iteration number using MRI image, (right) SNR vs iteration number using a phantom image, (Down)(left) SNR vs undersampling factor using MRI image, and (right) SNR vs undersampling factor using a phantom image

This section presents an experiment to study how the SNR varies with the number of undersampling masks, and the number of iterations (Fig. 7). This experiment was done because the major problem in low field MRI systems is low SNR. We demonstrated the significance of AS-DLMRI in improving the SNR of the low-field MRI systems that are currently under development. It was observed, as shown in Fig. 7 below, that the value SNR increases as the number of iterations increased both with AS-DLMRI and DLMRI, and it remained constant with LDP. A related experiment was also done to determine SNR with different values of undersampling masks in k-space, and it was observed that the values of SNR decreased as the value of undersampling masks increased in k-space. In both experiments, AS-DLMRI produced relatively higher values of SNR when compared to DLMRI and LDP.

### Computational cost

**Fig. 8:**
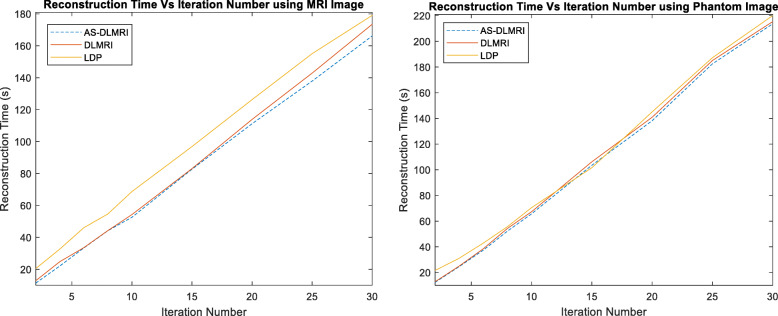
Algorithms’ Reconstruction time: (left) using MRI image, and (right) using phantom image

Determining the complexity of the algorithm with several dictionary variations is challenging [13]. It was also difficult for us to determine the actual complexity of AS-DLMRI but we explained the complexity of some of the operations. Nevertheless, experimental results reveal that AS-DLMRI is relatively faster than DLMRI. The operations that increase the complexity of our algorithm are:

1. The number of dictionary learning iterations, P. For the dictionary size to converge, both P and K should be large (in all our experiments, we used *P* ≥ 200 and k = 5).
2. The computation of ITC, which is computed for each dictionary’s representation. The overall operation is bounded by *(n*_*cand*_*s/n) N*, which depends on the number of signals present on each iteration and this operation happens only every k^th^ iteration, the extra complexity is relatively small. During the experiment, it was noted that AS-DLMRI required relatively low reconstruction time when compared to DLMRI and LDP. Figure 8 below shows the reconstruction time for each of the three algorithms.

## Discussions

In this section, we discuss the performance of AS-DLMRI in comparison to DLMRI and LDP. During this study, a brain MRI image obtained from [6] and a Phantom image that was acquired using low-field MRI at LUMC, were used in all our experiments. We used four performance metrics which include PSNR, SNR, HFEN and reconstruction time. These metrics were selected because they have been used in related studies. Also, all the experiments were done on a computer with no Graphical Processing Unit (GPU). In the results section, starting with reconstruction visual quality, it was observed that there was a slight improvement in reconstruction quality with AS-DLMRI when compared to DLMRI and LDP. However, there was only a small visual difference between AS-DLMRI and DLMRI. When compared to LDP, there was a significant difference in visual quality between AS-DLMRI and LDP. Comparing AS-DLMRI and DLMRI in terms of PSNR, AS-DLMRI had slightly higher values of PSNR. For LDP, the PSNR values remained almost constant as the number of iterations increased. For HFEN, the values reduced as the number of iterations increased in both AS-DLMRI and in DLMRI while the value remained constant with the number of iterations with LDP algorithm. The decrease in HFEN indicates that AS-DLMRI and DLMRI demonstrated superior performance in capturing edges and fine features during reconstruction. Experiments on various undersampling factors revealed that the values of PSNR reduced with the increase in number of undersampling factors while the values of HFEN increased with increasing undersampling factors with both AS-DLMRI and DLMRI. This means that lower values of undersampling masks in k-space resulted into a high PSNR value and lower value of HFEN resulting into high quality reconstructions. Experiments on SNR revealed that the value increased with the increase in the number of iterations in both AS-DLMRI and DLMRI but it remained constant with LDP. AS-DLMRI had higher SNR values when compared to DLMRI and LDP demonstrating its potential for addressing the issue in systems with low SNR problems like low-field MRI. Experiments on SNR with undersampling factors in k-space revealed a decrease of SNR with increase in the values of undersampling factors in all the algorithms but AS-DLMRI had a slightly higher values when compared to DLMRI and LDP. All the experiments demonstrated the potential of using AS-DLMRI on ordinary computers using a standard CPU and no GPU. Therefore, AS-DLMRI maybe suitable for use in low-field MRI and in low resource settings like Uganda where access to high-end computing hardware like Graphical Processing Units (GPUs) is still limited.

## Conclusions

Our proposed algorithm adapts the size of the dictionary that is suitable for the input signal. It has been noted in all our experiments that AS-DLMRI was slightly better in terms of PSNR, SNR and HFEN when compared to DLMRI and was significantly better than for LDP. Moreover, our proposed algorithm is also faster than DLMRI. Moreover, a steady increase of PSNR and SNR was obtained as the dictionary learning iterations increased, thereby concluding that AS-DLMRI adjusts well to our input signal. During this study, we compared our results with DLMRI and LDP. This is because these two algorithms have been used as a benchmark algorithm in related studies. Experimental results revealed that using an adaptive-size dictionary may help to reduce the computational complexity, and also to improve the quality of the reconstructed images since only relevant atoms are utilized during reconstruction. However, AS-DLMRI did not completely remove noise during the experiments with the noisy phantom. Therefore, integrating AS-DLMRI with an image denoising function may remove noise completely from noisy images that are common with Low-Field MRI systems. This will be the next step in our research.

## Data Availability

The data and the code used during this study will be shared on request by the corresponding author.

## Abbreviations

MRI: Magnetic resonance imaging
SNR: Signal-to-noise-ratio
CNR: Contrast-to-noise ratio
ML: Machine meaning
DL: Dictionary learning
PSNR: Peak Signal-to-Noise Ratio
CS: Compressed Sensing
GPUs: Graphical Processing Units
HFEN: High Frequency Error Norm.
